# Investment in roads and traffic safety: linked to economic development? A European comparison

**DOI:** 10.1007/s11356-022-22567-y

**Published:** 2022-08-22

**Authors:** José Navarro-Moreno, Francisco Calvo-Poyo, Juan de Oña

**Affiliations:** TRYSE Research Group, Department of Civil Engineering, University of Granada, ETSI Caminos, Canales y Puertos, Campus de Fuentenueva, s/n, 18071 Granada, Spain

**Keywords:** Investment in roads, Road maintenance expenditure, Road safety, Transport policy, Sustainable transportation

## Abstract

This study analyzes how economic resources invested in roads may affect mortality, depending on the level of economic development of a country. To this end, 23 European countries were classified into two groups—high-income countries and low-income countries—according to their average Gross Domestic Product (GDP) per capita over the period 1998–2016. The economic resources are considered through the investment in construction and the maintenance expenditure. Further variables are included to control for several factors related to the infrastructure, socioeconomics, legislation, and meteorology. Fixed-effects panel data models were built separately for the interurban road network of each group of countries. These models also capture the international inequalities within each group and the country-specific national trend for the study period. The main results indicate a reduction effect on the fatality rate of road maintenance expenditure (in both groups), and of the investment in construction (in the low-income countries). Other variables—such as proportion of motorways, motorization rate, unemployment rate, GDP per capita, alcohol consumption, Demerit Point System, and mean annual precipitation—showed statistically significant results as well. Finally, the country-specific fixed effects and the country-specific trend were mapped geographically, to better reflect national conditions for achieving lower fatality rates in the high-income countries, and greater progress in reducing fatalities in the low-income countries. In the end, this study provides evidence to policy-makers that can help to achieve a safer and more sustainable transport system, namely, how to tackle an ongoing major problem—traffic-related deaths—when attending and allocating the economic resources that road infrastructure needs.

## Introduction

Road fatalities continue to be a major public health problem worldwide and a key issue in most national transport policies. In fact, they have gone from being the eighth leading cause of death worldwide in 2007 to the sixth in 2017, with global figures of 1.2 million deaths per year (GBD 2017 Causes of Death Collaborators 2018). In addition to the suffering generated by these losses, there is also a high socioeconomic burden due to road traffic injuries. The social cost of road crashes may range on average from 1.1% to 2.9% of the GDP (Wijnen and Stipdonk 2016). Further knowledge of the factors behind such crashes will no doubt help us tackle this serious problem that affects public health, the economy, and society in general.

In the context of the European Union (EU), 18,786 people lost their lives as a consequence of road crashes in 2020 (Eurostat 2022). Although this figure marks a 37% decrease from 2010 figures, it should be interpreted in light of the circumstances of the COVID-19 pandemic and associated mobility restrictions in 2020. In fact, between 2019 and 2020 alone there was a 17% decrease in traffic deaths (ETSC 2021). In addition, this decade of slow reduction in road fatalities is marked by the financial crisis of 2008-2009 and the sovereign debt crisis in 2011–2012. This succession of crises has eroded the investments made by European countries in their road infrastructure. Spending on road maintenance has gone from the equivalent of 0.8% of EU GDP in 2008 to 0.5% in 2013, highlighting a maintenance backlog in countries such as Germany, the Netherlands, Italy, or Spain (European Commission 2018). Such data underline a need to explore the relationship between the economic resources invested in roads and the actual road fatalities. Given the heterogeneity of socioeconomic circumstances among EU countries, an added objective was to see whether these differences can further influence the effects of road investment on road fatalities.

In short, this study looks into the effects that economic resources invested in roads (along with other related factors) may have on mortality, in view of the economic level of a country and with a focus on the interurban road network.

The article is organized as follows: first, a review of the state of the art is offered in the “Literature review” section, considering international road safety studies that include socioeconomic variables, and those that incorporate variables of economic resources invested in road infrastructure. The “Methodology” section presents the methodology applied, while the “Results and discussion” section shows the results obtained and discusses them. Finally, the “Conclusions” section presents the main conclusions of this study.

## Literature review

### Studies at an international level

In analyses at the international level and with aggregate data, socioeconomic factors have been extensively studied in road safety literature. Among them, per capita income and unemployment rate are the most commonly used. Van Beeck et al. (Van Beeck et al. 2000) used per capita income to analyze the association between the level of prosperity of 21 countries of the Organisation for Economic Co-operation and Development (OECD) and fatal road crashes. The results showed that this association changed sign at a certain level of prosperity: up to that level, economic development was associated with an increase in road mortality, after which increased prosperity was associated with a decrease. Kopits and Cropper (Kopits and Cropper 2005) further explored the issue by analyzing a panel of 88 countries and distinguishing the geographic region to which they belong. In this way, and controlling for the specific time trend of each region, they found that the mortality rate—fatalities per population—began to decrease at a per capita income of $8,000 (1985 international dollars). Gerdtham and Ruhm (Gerdtham and Ruhm 2006) studied the relationship of macroeconomic conditions in 23 OECD countries with respect to various causes of death, including those caused by traffic crashes. They used the unemployment rate as a control for the labor market situation. As a main result, they found that a 1% decrease in unemployment would lead to a 2.1% increase in the number of road deaths.

Bishai et al. (Bishai et al. 2006) studied the influence of GDP per capita on the road safety of 41 countries grouped into “lower income countries” and “wealthy countries.” They found that a 10% increase in GDP in a lower income country would lead to a 7.9% increase in crashes, 4.7% more injuries, and 3.1% more fatalities. Contrariwise, the increase in GDP in the case of wealthier countries could be associated with a reduction in the number of deaths, but not with the number of crashes or injuries. Subsequently, Gaygisiz (2009) added to the economic indicators already mentioned—GDP per capita and unemployment—the Gini index and a series of variables related to cultural characteristics. This author arrived at different qualitative associations. For example, countries with a high accident rate were associated with a greater acceptance of social inequalities, while countries with a low rate showed greater individualism. A relationship also appeared between favorable economic conditions (high per capita income, low unemployment, and low income inequality) and higher road safety.

Along these lines, numerous studies have considered socioeconomic factors in explaining road accident rates. Elvik (Elvik 2015) conducted a review of 20 studies to analyze the mechanisms through which such factors influence road safety. In doing so, he formulated a model for 14 OECD countries and was able to link the 2009-2010 recession to a reduction of 4,850 fatalities in the selected set of countries. Yaseen et al. (Yaseen et al. 2018) extended the scope of study to 30 OECD countries and explored the short- and long-term associations of various socioeconomic and environmental factors with road traffic fatalities. As the most significant result, the authors arrived at a 0.947% reduction in road traffic fatalities for every 1% increase in health expenditures. Ali et al. (2019a) conducted a continent-by-continent comparison of the possible factors (mainly socioeconomic) associated with the fatality figures of 42 high-income countries. To do so, they divided the countries into three panels—Asia, Europe, and America—and calculated the elasticities of the different factors. The results showed diverse relationships according to the temporal impact and geographical scope. Thus, an increase in GDP per capita was associated in the long-term with a decrease in deaths in Europe and America, while in the short-term, the opposite was true for Asia and Europe. In a related study, Ali et al. (Ali et al. 2019b) divided a total of 27 upper middle income countries by continent (Asia, Europe, and America). In this case, however, the increase in GDP per capita was associated with an increase in mortality on all three continents, both in the short- and long-term. On the other hand, the increase in health expenditure appeared to be associated with a long-term reduction in deaths.

In the case of macro panels at the European level, the variables of unemployment and per capita income are again mainly used to control for the socioeconomic situation of a country. Economou et al. (Economou et al. 2008) conducted a study on the relationship between unemployment and ten causes of death for 13 European countries—in the wake of the analysis conducted by Ruhm (Ruhm 2000) for the case of the USA. Unlike Ruhm (Ruhm 2000), they found a direct relationship between adverse economic conditions and mortality. However, in the case for the number of traffic fatalities, the figures also declined when the unemployment rate increased. In turn, Yannis et al. (Yannis et al. 2011) used two socioeconomic indicators —motorization rate and population— to model the trend in fatality figures for eight EU countries and identify the points marking a change in trend. Castillo-Manzano et al. (2014) used, in addition to GDP per capita and motorization rate, a series of socioeconomic variables related to the health systems of 27 EU countries. They report evidence of how hospital bed density and public expenditure may affect healthcare in terms of reduced road deaths, just as GDP per capita and motorization rate showed beneficial effects on road safety.

Deepening into the relationship between socioeconomic aspects and mortality, Antoniou et al. (2016) analyzed the GDP and the number of fatalities of 30 European countries by means of long-term time-series models. Nikolaou and Dimitriou (2018) used GDP as well as other socioeconomic and demographic factors to assess the level of road safety of 23 EU countries concerning their stated objectives. They moreover established four clusters of countries and identified best-performing and under-performing countries within each cluster.

The analysis of the influence of a given factor for different groups of countries can prove very useful when establishing comparisons. Castillo-Manzano et al. (2020) used various socioeconomic indicators of the 28 EU countries to determine whether financial intervention carried out by the so-called Troika (European Commission, European Central bank, and International Monetary Fund) in five of them—Cyprus, Greece, Ireland, Portugal, and Spain—had any effect on accident and fatality figures. They concluded that the financial intervention did not bear an impact on road safety in these five countries. Stickley et al. (2021) studied educational inequalities and macroeconomic changes during the 2000s in the Baltic countries (Estonia, Latvia, and Lithuania) and Finland. They observed that the number of deaths decreased more in the Baltic countries than in Finland. The impact of the economic recession was also accentuated in the Baltic countries. Finally, they pointed out that investment in roads and access to better cars could influence the reduction of mortality.

### Studies that consider the economic resources invested in roads

Some studies incorporate economic resources invested in roads as an explanatory factor for road accident rates, although most do so for a single-country model: Norway (Fridstrøm and Ingebrigtsen 1991), New Zealand (Guria 1999), Spain (Albalate et al. 2013; Aparicio Izquierdo et al. 2013; Rojo et al. 2018; Sánchez González et al. 2020; Sánchez González et al. 2018), the USA (Nguyen-Hoang and Yeung 2014), China (Sun et al. 2019), and Chile (Sánchez-González et al. 2021). Among these studies, the ones that calculate expenditure on road maintenance show overall consistency as to its reducing effect on casualty figures (Albalate et al. 2013; Fridstrøm and Ingebrigtsen 1991; Nguyen-Hoang and Yeung 2014; Rojo et al. 2018; Sánchez González et al. 2018). Still, investment in road construction may show different signs depending on several factors: ownership of the road (Fridstrøm and Ingebrigtsen 1991), country considered in the study (Sánchez González et al. 2018; Sun et al. 2019), its definition as an increase in capital value (Nguyen-Hoang and Yeung 2014) or economic level of the region receiving the investment (Sánchez González et al. 2020). Sánchez González et al. (2020) found that the economic resources invested in roads showed different effects—even within the same country—according to the per capita income of the region considered. An international study by Calvo-Poyo et al. (2020) considered both investment in road construction and maintenance expenditure for 23 European countries. In view of the results, the authors highlighted the role of maintenance expenditure, together with socioeconomic variables such as motorization rate and GDP, in reducing the mortality rate. Conversely, investment in road construction showed a direct relationship with mortality.

In sum, the influence of various socioeconomic aspects—but particularly GDP per capita and unemployment rate—on road safety has been analyzed in numerous studies at the international level. It has been shown that the level of economic development of a country or region can indeed influence the sign or magnitude of this relationship. Notwithstanding, the literature lacks references that analyze, at an international level, the influence of economic resources invested in roads on road mortality according to a country’s economic development. Given the large socioeconomic differences among European countries, investment in road construction and maintenance expenditure may bear a different impact on road mortality depending on the level of economic development. This article specifically distinguishes between two groups: high-income and low-income countries.

## Methodology

### Selection of variables and data sources

In the present study, a mortality rate defined as the number of fatalities per billion passenger-km is used as the dependent variable. The choice of this variable was based on the availability of data at the European level, on the analysis of the suitability of the various risk indicators (Farchi et al. 2006; Papadimitriou et al. 2013), and on the recommendation by Hakkert and Braimaister (2002) to use a ratio depending on the level of exposure.

The target independent variables of this article are the economic resources invested in roads, in terms of both construction and maintenance. To make comparisons, the two variables are expressed as a unitary one, dividing the investment in construction and the expenditure on maintenance by the extension of the road network in each country, as done in previous studies (Fridstrøm and Ingebrigtsen 1991; Aparicio Izquierdo et al. 2013; Sánchez González et al. 2020; Sánchez González et al. 2018; Calvo-Poyo et al. 2020; Sánchez-González et al. 2021).

Regarding the rest of the independent variables considered to explain the study problem, GDP per capita, unemployment rate, and motorization rate are included because they are the most recurrent ones in the literature analyzed (Antoniou et al. 2016; Bishai et al. 2006; Castillo-Manzano et al. 2021; Castillo-Manzano et al. 2020; Economou et al. 2008; Elvik 2015; Gaygisiz 2009; Gerdtham and Ruhm 2006; Kopits and Cropper 2005; Nikolaou and Dimitriou 2018; Ruhm 2000; van Beeck et al. 2000; Yannis et al. 2011).

In addition, control variables less frequently represented in road safety models are included here to better explain the context of study. These variables are as follows: alcohol consumption, the implementation of a Demerit Point System (DPS), and mean precipitation. Because over 20% of fatalities worldwide are alcohol-related (Vissers et al. 2017), the variable of alcohol consumption per capita merited consideration, albeit indirectly, since the data are for the total population and not only drivers. The implementation of some point-based license system is included because the effectiveness of this type of legislative measure has been demonstrated in several studies (Abay 2018; Castillo-Manzano and Castro-Nuño 2012; De Paola et al. 2013; Izquierdo et al. 2011; Lee et al. 2018; Zambon et al. 2007). Finally, mean precipitation is the most widely used meteorological parameter in road safety studies (Aparicio Izquierdo et al. 2013; Bergel-Hayat et al. 2013; Calvo-Poyo et al. 2020; Eisenberg 2004; Fridstrøm et al. 1995; Fridstrøm and Ingebrigtsen 1991; Nguyen-Hoang and Yeung 2014; Sánchez-González et al. 2021; Sánchez González et al. 2020; Sánchez González et al. 2018; Theofilatos and Yannis 2014). Mean annual precipitation is therefore included to approximately account for the meteorology of each country. The definition of the selected variables, as well as the data source for each, are shown in Table 1.

**Table 1:** Definition of variables and data source

Variable	Definition	Data Source
Fatalities	Fatalities per billion pkm	UNECE; CARE; IRTAD; *Ministerstvo-Vnútra, Slovenia;* EU’s DG Mobility and Transport.
Road_inv /Road_maint	Road construction investment/road maintenance expenditure, in thousand euros per km (2015 prices)	OECD/ITF; *Ministerstvo Dopravy*, Czechia; *Bundesministerium für Verkehr und digitale Infrastruktur*, Germany; *Ministerie van Infrastructuur en Waterstaat*, Netherlands; *Ministério da Economia e Transição Digital and Infraestruturas de Portugal*, Portugal; *Ministerio de Transportes, Movilidad y Agenda Urbana,* Spain; *Trafikverket*, Sweden.
Prop_motorwa	Proportion of motorways over the total road network (%)	Eurostat; EU’s DG Mobility and Transport
Mot_index	Motorization index, in cars per 1000 inhabitants	Eurostat; EU’s DG Mobility and Transport; *Bundesministerium für Verkehr und digitale Infrastruktur*, Germany
Unemploy	Unemployment rate (%)	Eurostat; World Bank
GDP_Cap	GDP per capita, in thousand euros per inhabitant (2015 prices)	World Bank; OECD
Alcohol	Alcohol consumption, in liters per capita (age > 15)	World Health Organization
DPS	Demerit Point System, dummy variable (0: no; 1: yes)	European Transport Safety Council; Klipp et al. (2011)
Precipit	Average depth of rain water during a year (mm)	Copernicus Climate Change Service (2018)

To account for country-specific characteristics not addressed through the selected variables, but that may influence road mortality, a series of dummy variables were added. Moreover, by controlling for country-specific linear trends, as in previous studies (Castillo-Manzano et al. 2014; Kopits and Cropper 2005; Nguyen-Hoang and Yeung 2014), possible effects of unconsidered trends (such as technological changes in vehicles or driver behavior, for example) that vary over time could be captured.

### Definition of country grouping and description of data

To form the two groups of countries to be analyzed—called high-income countries (HIC) and low-income countries (LIC)—the GDP per capita of each country was calculated as constant 2015 prices for each year during the period 1998-2016, following (Sánchez González et al. 2020). From these figures, the average value of the series for each country was calculated to then arrive at the global average value: 30,316.33 €. This figure served to classify the 12 countries having a higher GDP per capita as HIC, and the remaining 11 as LIC. The countries included in each group are shown in Table 2.

**Table 2:** Country grouping

High-income countries (HIC)	*Austria, Belgium, Denmark, Finland, France, Germany, Ireland, Luxembourg, Netherlands, Norway, Sweden, and United Kingdom*
Low-income countries (LIC)	*Croatia, Czechia, Estonia, Italy, Latvia, Lithuania, Poland, Portugal, Slovakia, Slovenia, and Spain*

**Fig. 1:**
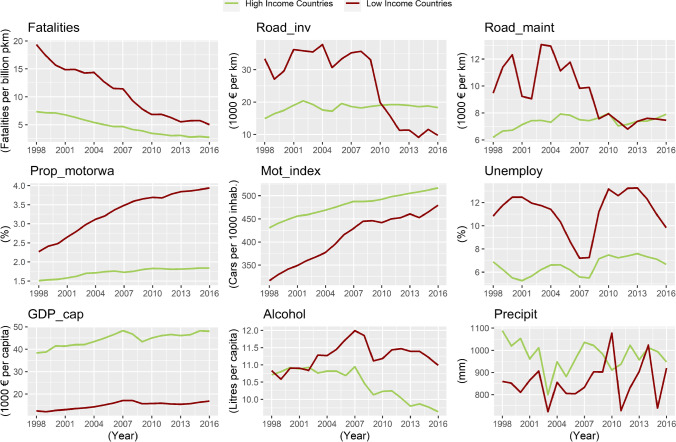
Evolution of the mean values of the variables

Once the countries had been classified, the main descriptive statistics of the variables for each group of countries were derived, shown in Table 3. This grouping already evidences some noteworthy differences between the HIC and LIC statistics, as seen in Fig. 1.

**Table 3:** Descriptive statistics

	High-income countries				Low-income countries			
Variable	Mean	Std. dev.	Min	Max	Mean	Std. dev.	Min	Max
Fatalities	4.72	2.35	1.47	12.39	10.82	6.75	2.58	35.3
Road_inv	18.36	18.12	1.13	101.35	25.87	46.87	0.03	279.89
Road_maint	7.35	5.11	0.25	21.5	9.46	14.39	0.47	83.76
Prop_motor	1.76	1.60	0.11	5.65	3.30	5.30	0.00	21.42
Mot_index	480.04	75.06	324.07	678.41	409.25	106.08	199.39	625.17
Unemploy	6.54	2.40	1.90	15.50	11.20	4.40	4.00	26.10
GDP_cap	44.40	15.00	30.18	94.85	14.96	6.72	4.27	31.74
Alcohol	10.45	2.20	5.24	14.95	11.25	2.34	6.35	17.75
DPS	0.36	0.481	0	1	0.239	0.43	0	1
Precipit	976.08	230.01	520.23	1548.14	860.33	316.59	445.70	2266.78

Hence, the mean mortality rate in LICs is more than double that in HICs (Table 3). Yet, as can be seen in Fig. 1, there is a generalized decrease and convergence in the rates throughout the study period.

Investment in road construction was 41% higher in the LICs during the study period (Table 3). This can be explained by the fact that, due to their infrastructure deficit, the LICs had better access to various EU funding mechanisms (Cohesion Policy Funds), even from the pre-accession phase to the EU. Specifically, during the period 2000-2013, the EU transferred €64,681 million through cohesion policy funds, mostly to countries belonging to the LIC group (European Court of Auditors 2013). Figure 1 shows that investment in road construction up to 2010 is much higher in LICs than in HICs; and while in HICs, the investment per kilometer remains relatively stable at around €20,000, in LICs, it is around €35,000. In contrast, the 2008 financial crisis particularly affected LICs, whose unit investment fell from €35,631 in 2008 to €9,695 in 2016. The average maintenance expenditure is 29% higher in LICs (Table 3), yet a look at the evolution of this variable reveals that maintenance expenditure is higher in LICs until 2008, most likely for the very reason given above. From 2009 onwards, both groups of countries show an expenditure close to €7,500 per kilometer of network, and then continue with very similar values.

The higher proportion of motorways in LICs (87% more according to Table 3) is due to their massive construction and the widening of conventional roads during the study period (Fig. 1), interventions mainly financed by the Cohesion Policy Funds. These countries previously had a less extensive road network, meaning that the proportion of motorways increased significantly in this group of countries during the study period (Schipper 2008).

The motorization rate is 17% higher in the HICs (Table 3), being largely related to the per capita income of each country (Kopits and Cropper 2005; Medlock and Soligo 2002). Over the study period, it increases in both groups of countries, although a converging trend is seen. Growth is greater in the LICs, possibly owing to the fact that they started from lower motorization rates and to their overall economic development during the study period. Thus, in 1998, the motorization rate of HICs exceeded that of LICs by 114 units, whereas in 2016, the difference between groups was reduced to 37 units.

The unemployment rate is almost twice as high in LICs as in HICs (Table 3). Figure 1 shows that this variable is very sensitive to economic crises, especially in LICs. Until 2004, the variable shows opposite trends between HICs and LICs. From then on, the trend follows a similar behavior in both groups, notwithstanding greater inequality in the wake of the economic crisis of 2008. Accordingly, LICs increase their unemployment rate by 5.97% between 2008 and 2013, while HICs witness an increase of just 2.11% for the same period.

As seen in Table 3, the per capita income of HICs is nearly 3 times that of LICs. Both HICs and LICs reach their maximum value in 2007: 48,275 € for HICs and 17,139 € for LICs (Fig. 1). After the economic crisis of 2008, both groups undergo a reduction in those figures. A decreasing trend begins in LICs and maintains values close to the 2009 minimum (with recovery from 2014 onwards). This negative trend is reversed in HICs from 2009, though the per capita income level of 2007 is not recovered.

Regarding per capita alcohol consumption, Table 3 shows similar values for the two groups. However, when looking at the evolution of average consumption in Fig. 1, behavior appears to depend on the group of countries. Both groups have similar values until 2007, but thereafter, alcohol consumption in HICs gradually decreases until 2016. In contrast, in LICs, there is a large increase in consumption until 2007 and a drop with the arrival of the economic crisis—although this drop is not maintained (as in HIC) and there is a later upturn in alcohol consumption.

HICs show a higher value in the dataset concerning mean annual precipitation: 976.08 mm for HICs, and 860.33 for LICs (Table 3). It should be noted that the grouping of countries according to per capita income includes different climatic regions. Even so, both the mean and its evolution appear to be consistent with the grouping of countries, since the HICs (including countries in central and northern Europe) have more precipitation than the LICs (most countries in southern, eastern, and western Europe).

Finally, the dummy variable DPS reflects the introduction of a point-based licensing system during the period considered. Of the HICs, six countries adopted some form of DPS—Austria (2005), Denmark (2005), Ireland (2002), Luxembourg (2002), Netherlands (2002), and Norway (2004)—and among the LICs, just five—Italy (2003), Czechia (2006), Latvia (2004), Portugal (2016), and Spain (2006).

### Data checks and processing

For the variables used as regressors, no multicollinearity problems were detected. The correlations between each pair of variables were verified, none of them showing a coefficient higher than 0.8. It was also verified whether the joint Variance Inflation Factor (VIF) in the two groups of countries exceeded a value of 10, a figure generally considered as the threshold indicative of this “problem” (Gujarati 2003). The VIF is 8.72 for HICs and 4.92 for the LICs, meaning the threshold is not reached. In addition, to ensure the stationarity of the different time series, the existence of unit roots in the panels was checked. For this purpose, and due to the existence of contemporaneous correlation, the Breitung test (Breitung and Das 2005) was applied. This test assumes, in the null hypothesis, the existence of unit roots, and in the alternative hypothesis, the stationarity of the panels. The test results strongly accept the null hypothesis for the variables of proportion of high-capacity roads and motorization rate, and to a lesser extent, for per capita income and alcohol consumption. So as to achieve stationarity of these four variables, we differentiated them, considering their annual variation in the model.

### Formulation of the model

Given the nature of the data, with two groups of countries and covering 19 years, it was decided to formulate separate panel data models with fixed effects by country and time trend. The inclusion of fixed effects allows for controlling and quantifying time-invariant country-specific omitted variables, while the inclusion of the trend would serve to identify the different national trends. The objective is to explain the road fatality rate over time and in the selected countries within each group, and to compare them with each other. Such models are widely used in road literature at the macro level. Still, they entail several particular aspects of their own that must be analyzed to obtain robust estimators. To this end, a series of tests were carried out to verify the hypotheses of homoscedasticity across cross-sections (Levene (1960)), non-existence of first-order serial correlation (Wooldridge (2007)) and cross-section independence (test de Pesaran (2020)). The results of these tests, in which the null hypothesis assumes the aforementioned properties, are strongly rejected, as can be seen in Table 4. Consequently, the existence of groupwise heteroscedasticity, first-order autocorrelation, and cross-sectional dependence is considered.

**Table 4:** Test of homoscedasticity, serial correlation, and cross-sectional dependence

	High-income countries		Low-income countries	
Levene test	W0: F (10, 198)	15.0477	W0: F (11, 216)	7.959
	Prob. > F =	0.000	Prob. > F =	0.000
	W50: F (10, 198)	8.508	W50: F (11, 216)	5.483
	Prob. > F =	0.000	Prob. > F =	0.000
	W10: F (10, 198)	14.467	W10: F (11, 216)	7.556
	Prob. > F =	0.000	Prob. > F =	0.000
Wooldridge test	F (1, 10) = Prob. > F =	126.202 0.000	F (1, 11) = Prob. > F =	6.815 0.024
Pesaran test	Value =	22.799	Value =	14.768
	Prob. =	0.000	Prob. =	0.000
	Absolute mean value of the residual correlation =	0.725	Absolute mean value of the residual correlation =	0.535

To solve these problems, the GLS-Parks estimator (Parks 1967) is used, which considers groupwise heteroscedasticity, autocorrelation, and cross-sectional dependence in its error structure. Authors Reed and Webb (2010) pointed out that for a ratio between the number of years of study, T, and number of countries considered, N, greater than 1.5, the Parks estimator is the most efficient. In the present study, the ratio is 1.64 for LIC and 1.5 for HIC. Thus, the panel data model used takes the following form:

$$y_{\mathrm i\mathrm t}=a_i+\beta_{\mathrm K}X_{\mathrm K\mathrm{it}}+\tau_{\mathrm i}T_{\mathrm i\mathrm t}+\mu_{\mathrm i\mathrm t}$$

in which *y*_it_ represents the explained variable, with subscripts *i* for each country and *t* for each year, *𝛼*_*i*_ are dummy variables per country, *X*_Kit_ are the explanatory variables, *β*_K_ are their estimable coefficients, *T*_t_ is the country-specific linear trend, 𝜏_i_ the slope of that trend, and *μ*_it_ is the following error term:

$${\mu}_{\mathrm{i}\mathrm{t}}={\rho}_{\mathrm{i}}{\mu}_{\mathrm{i}\mathrm{t}-1}+{e}_{\mathrm{i}\mathrm{t}}$$

where *ρ*_i_ is the country-specific autocorrelation parameter and *e*_it_ corresponds to the independent and identically distributed errors.

However, as stated by Beck and Katz (1995) and later confirmed by other studies (Moundigbaye et al. 2018; Reed and Webb 2010), the GLS-Parks method produces unrealistic standard errors, which leads to rejecting hypotheses too easily. This may invalidate statistical inference based on such standard errors. The fixed effects model with Driscoll and Kraay (1998) standard errors and lag length-selection using Newey and West’s plug-in procedure (Newey and West 1994) will also be used to obtain errors robust to groupwise heteroscedasticity, cross-sectional dependence, and autocorrelation (Hoechle 2007). The software used to estimate the models is STATA version 12.1.

## Results and discussion

The models obtained from the available data are shown in two separate tables: Table 5 shows the models for HICs, while Table 6 shows the models for LICs.

**Table 5:** Results for high-income countries

High-income countries				
	(1)	(2)	(3)	(4)
	GLS-Parks	GLS-Parks	GLS-Parks	GLS Driscoll-Kraay SE
Road_inv	.006	.005	.01**	.019*
	(.006)	(.006)	(.005)	(.011)
L1. Road_inv	.008	.027***	.002	**−**.005
	(.006)	(.006)	(.005)	(.011)
Road_maint	−.056***	−.107***	**−**.012	.016
	(.013)	(.014)	(.012)	(.027)
L1. Road_maint	−.075***	−.11***	−.02*	−.053**
	(.014)	(.014)	(.01)	(.024)
D.Prop_motorwa		.092	−.316***	**−**.447
		(.203)	(.102)	(.608)
D.Mot_index		.01***	.003***	.003
		(.002)	(.001)	(.002)
Unemploy		−.25***	−.121***	−.115***
		(.023)	(.015)	(.023)
D.GDP_Cap		.008	**−**.006	**−**.001
		(.006)	(.004)	(.011)
D.Alcohol		.159***	.139***	.17***
		(.021)	(.015)	(.048)
DPS		−1.048***	−.804***	−1.012***
		(.097)	(.075)	(.189)
Precipit		0	0	0
		(0)	(0)	(0)
_cons	5.127***	7.549***	4.531***	4.329***
	(.331)	(.301)	(.232)	(.736)
Observations	216	216	216	216
*R*^2^ (within)	-	-	-	0.9625
Country FE	No	No	Yes	Yes
Country Trend	No	No	Yes	Yes

Standard errors are in parentheses. “L1” means 1-year lag and “D” means that the series are differentiated

***p<.01, **p<.05, *p<.1

**Table 6:** Results for low-income countries

Low-income countries				
	(1)	(2)	(3)	(4)
	GLS-Parks	GLS-Parks	GLS-Parks	GLS Driscoll-Kraay SE
Road_inv	−.003	−.009**	−.011***	−.017***
	(.003)	(.004)	(.002)	(.004)
L1. Road_inv	.006**	.009**	.003	−.007
	(.01)	(.004)	(.002)	(.005)
Road_maint	−.033***	−.02**	0	−.002
	(.009)	(.008)	(.006)	(.013)
L1. Road_maint	−.029***	−.048***	**−**.02***	−.022**
	(.01)	(.008)	(.006)	(.01)
D.Prop_motorwa		−.567**	−.614***	−.83**
		(.256)	(.184)	(.349)
D.mot_index_1000		.001	−.003	.002
		(.003)	(.003)	(.005)
Unemploy		−.025	−.133***	−.188**
		(.035)	(.028)	(.066)
D.GDP_Cap		.196**	.301***	.473**
		(.095)	(.07)	(.173)
D.Alcohol		.189***	−.053	−.14
		(.07)	(.045)	(.082)
DPS		−.809***	−.228	−.653
		(.246)	(.187)	(1.163)
Precipit		−.003***	−.001***	−.001*
		(0)	(0)	(.001)
_cons	9.794***	11.921***	9.448***	11.589***
	(.719)	(.707)	(.711)	(1.456)
Observations	198	198	198	198
*R*^2^ (within)	-	-	-	0.9464
Country FE	No	No	Yes	Yes
Country trend	No	No	Yes	Yes

Standard errors are in parentheses. “L1” means 1-year lag and “D” means that the series are differentiated.

***p<.01, **p<.05, *p<.1

### Models for HICs

To check for possible changes in the model coefficients and to avoid omitted variable bias, the independent variables were added one by one, following a stepwise algorithm. An extended version of this process is shown in the appendix (Table 8).

The models obtained for the HICs show that the significant variables do not change sign with the incorporation of new independent variables or with the inclusion of fixed effects by country and year. In model (1), which includes just the target variables, only the maintenance expenditure variable is significant (both in the year in which the expenditure is made and during the year after). This variable shows an inverse relationship with the dependent variable, indicating that spending on maintenance contributes to reducing the number of deaths in road crashes, both in the year in which the expenditure is made and in the subsequent year.

When incorporating the rest of the independent variables in model (2), investment in road construction with a 1-year lag begins to show a significant relationship with a positive sign. Regarding the rest of the variables, the annual change in the motorization rate and the annual change in alcohol consumption are significant with a positive sign (direct relationship with mortality). The unemployment rate and the incorporation of a Demerit Point System are also significant, but with a negative sign (inverse relationship with mortality). The rest of the independent variables incorporated in the model (annual variation in the proportion of motorways, annual variation in per capita income, and mean precipitation) are not significant.

Next, in order to identify the specific characteristics of each country and to control for the specific trend that each country followed during the study period, fixed effects and the linear trend by country were incorporated in models (3) and (4). In addition, an equivalent fixed effects model with robust Driscoll-Kraay errors is used in model (4) to guarantee statistical inference and to be able to draw conclusions regarding the values of the estimated coefficients, the fixed effects, and the country-specific trend. The fixed effects and country-specific trend of model (4) are those represented geographically in section 4.4, while the numerical values of the corresponding fixed and trend effects of both models (3) and (4) can be found in the appendix (Table 8).

With respect to model (3), the main differences from model (2) are the appearance of a significant direct relationship for the investment in construction variable, and the loss of significance of investment with a 1-year lag, and of maintenance expenditure. In addition, the annual change in the proportion of high-capacity roads acquires a significant relationship with a negative sign. By using robust errors in model (4), the proportion of high-capacity roads and the motorization rate lose statistical significance. In model (4), the change in the standard errors can be seen, supporting comments in the literature that Parks' method tends to produce overly optimistic standard errors in the presence of contemporaneous correlation (Beck and Katz 1995; Hoechle 2007). As a consequence, the variables of proportion of high-capacity roads and motorization rate lose significance in model (4). On the other hand, and although the sign of the relationship does not change, the level of significance of the variables corresponding to the economic resources invested in roads varies slightly, now being 0.1 for investment in construction and 0.05 for expenditure on maintenance. The rest of the variables remain practically unchanged.

### Models for LICs

Continuing with the models for the case of LICs (Table 6), in model (1), investment in construction is significant and with a positive sign, only during the 1-year lag. Maintenance expenditure again shows significant results with a negative sign, both in the current year and for the 1-year lag. When incorporating the rest of the independent variables in model (2), investment in road construction begins to show a mortality-reducing effect during the year of execution. Similarly, the annual change in the proportion of high-capacity roads, the introduction of a DPS, and average annual precipitation also show this effect. In contrast, annual variations in GDP per capita and alcohol consumption reflect a direct effect on mortality increase.

As for the HICs, in models (3) and (4), we add the fixed effects and the country-specific trend, whose values can be consulted in the appendix (Table 9). Regarding the independent variables, model (3) shows that road construction (during the current year) and maintenance (with a 1-year delay) have an inverse relationship with the fatality rate. Among the significant variables, the proportion of high-capacity roads and the unemployment rate show an inverse relationship, and per capita income a direct relationship, while alcohol consumption and DPS lose significance. Then, in model (4), we note a considerable increase in the standard errors, as was expected and occurred in the case of the HICs. As a result, the variables that obtained significant results in model (3) decrease in significance—except for investment in construction, which remains at 0.01—although all of them remain significant results.

### Comparison between HICs and LICs

As previously mentioned, model (4) is the one that provides the best statistical inference, for which reason it is used to compare the results for both groups of countries (see Table 7). By comparing these results, it is possible to appreciate differences in the effects caused by the variables considered. Especially noteworthy are the effects of investment in construction and maintenance expenditure on the mortality rate.

**Table 7:** Comparison of models between HIC and LIC

	High-income countries	Low-income countries
Investment in road construction	.019*	−.017***
Investment in road construction (1-year lagged)	−.005	−.007
Expense on road maintenance	.016	−.002
Expense on road maintenance (1-year lagged)	−.053**	−.022**
Proportion of motorways (annual variation)	−.447	−.83**
Motorization rate (annual variation)	.003	.002
Unemployment rate	−.115***	−.188**
GDP per capita (annual variation)	−.001	.473**
Alcohol consumption (annual variation)	.17***	−.14
Demerit Point System	−1.012***	−.653
Mean annual precipitation	.000	−.001*

***p<.01, **p<.05, *p<.1

#### Investment in road construction (Road_inv)

Investment in road construction during the current year shows an inverse relationship with the mortality rate in LICs, while it shows a direct relationship (although much less significant) in HICs. This direct relationship between road construction investment and mortality found in the case of HICs was already reflected in aggregate studies at the European level (Calvo-Poyo et al. 2020), and in other previous studies at a national level (Fridstrøm and Ingebrigtsen 1991; Sánchez González et al. 2020, 2018). However, when considering a grouping of countries according to their economic development, an inverse relationship appears between investment in construction and mortality rate in the group of LICs. In the context of a country with low per capita income, this effect of construction investment has similarly been observed in a study conducted in China (Sun et al. 2019). In the case of Spain, Sánchez González et al. (2020) likewise found this inverse relationship for provinces with lower per capita income. Thus, in addition to the improvements in terms of accessibility and territorial cohesion that road investments bring to LICs, the present study shows that they contribute to reduction of the mortality rate. This beneficial effect on road safety may be due to the great improvement brought about by the construction of new high-capacity roads and the dualling of two-lane roads, carried out in these countries thanks largely to the European Cohesion Funds. It also should be noted that the fatality rate on high-capacity roads is much lower than on two-lane ones (Elvik and Vaa 2009; European Commission 2017).

#### Expense on road maintenance (Road_maint)

Regarding maintenance expenditure, the results for HICs and LICs coincide in terms of the reductive effect—though greater in HICs than in LICs—of this variable on the mortality rate during the year after the expenditure was made, as well as in the level of significance of the variable. This finding agrees with those reported at national levels (Albalate et al. 2013; Fridstrøm and Ingebrigtsen 1991; Nguyen-Hoang and Yeung 2014; Sánchez González et al. 2018).

Such results underline that adequate expenditure on road maintenance serves not only to avoid a 3 to 6 times higher cost in repair or renovation in the medium- to long-term (European Commission 2018), but can also contribute to the reduction of fatalities in the short-term.

#### Proportion of motorways (Prop_motorwa)

As regards the annual variation in the proportion of high-capacity motorways, the results show a significant influence with a negative sign in LICs, while for HICs the influence is lower and not statistically significant. Thus, the extension of the 11,856 km of motorways carried out in LICs during the period 2000–2016 would have led to improvements in mortality rates in these countries, in line with the results of studies at the European level (Albalate 2008; Calvo-Poyo et al. 2020; Castillo-Manzano et al. 2015, 2014). As for HICs, despite increasing their high-capacity network by 7,437 km during the same period, mortality reduction results do not appear immediately.

#### Motorization rate (Mot_index)

The annual variation of the motorization rate did not give significant results in either group of countries. Some studies show that an increase in the motorization rate leads to higher accident rates (Page 2001). Ali et al. (2019a) found that a 1% increase in the motorization rate is associated with increases in the number of fatalities in a selection of high-income countries in Asia and America (0.617% and 1.705%, respectively) but did not obtain conclusive results for European HICs. It has moreover been shown that when a certain threshold is reached, the trend reverses and the motorization rate becomes associated with reductions in mortality (Yannis et al. 2011). This situation occurs in countries featuring greater economic development and where more is invested in road safety, both at the individual and governmental levels (Kopits and Cropper 2005). In the present study, however, when considering the annual variation of the motorization rate, none of the effects specified above was reflected in the short-term that the variable represents.

#### Unemployment rate (Unemploy)

The unemployment rate does show significant results, with considerable differences between HICs and LICs. Specifically, the influence of unemployment on the reduction of mortality is 60% greater for LICs. It may be that the population at the highest risk of suffering a fatal accident, i.e. young people, is particularly affected by the increase in unemployment (Elvik 2015), which in turn decreases the use of private vehicles by this sector of the population. Such a trend would be accentuated in the case of LICs. Dietrich and Möller (2016) found that the young population is the most affected during adverse economic periods, as occurs from 2008 onwards within the study period.

#### GDP per capita (GDP_cap)

In the case of annual variation of GDP per capita, significant results (with a positive sign) were obtained only for LICs. To explain this result, the study by Yannis et al. (2014) can be evoked: in terms of macroeconomic dynamics, in the short-term, an increase in GDP per capita can be expected to have a direct influence on the increase in mortality, while in the long-term such an increase is a mortality-reducing factor (Yannis et al. 2014). Similar results were obtained by Ali et al. (2019). Such a beneficial effect is not manifest for HICs in the short-term. Even so, the medium/long-term beneficial effect of higher per capita income could be reflected in HICs through the lower fixed-effects coefficients of this group of countries, as will be discussed below.

#### Alcohol consumption (Alcohol)

Annual variation in per capita alcohol consumption is only significant among the HICs. Some LICs may reflect a remarkable situation involving a special influence. That is, countries such as Estonia and Lithuania underwent the largest increase in alcohol consumption between 1998 and 2016 (respectively from 8 and 6.3 to 15.4 and 13.6 liters per capita) while also being among the countries to show the strongest annual decreasing trend in the number of fatalities (see appendix, Tables 10, 11, 12). In addition, there is a particularly restrictive tradition regarding the maximum Blood Alcohol Content (BAC) allowed in the eastern European countries—six of the LICs set a maximum rate of 0.0 g/l for novice and commercial drivers, while in the HIC group, only Germany sets these levels (European Commission 2015). Finally, considerable differences are detected between HIC and LIC countries with respect to the percentage of serious injury accidents in which the blood alcohol level exceeded 0.1 g/l, ranging from 17.7% in Lithuania to 42.5% in Belgium (Legrand et al. 2013).

#### Demerit Point System (DPS)

Similarly, the DPS variable is found to be significant, with a negative sign only in HICs. Related to this result, several studies demonstrate the effectiveness of DPS in countries belonging to the LIC group (De Paola et al. 2013; Izquierdo et al. 2011; Zambon et al. 2007). Yet it has also been shown that the deterrent effect of these measures is limited over time (Abay 2018). Castillo-Manzano and Castro-Nuño (2012) quantified that the initial mortality reduction impact (around 15–20%) disappears in less than 18 months. As a consequence of this short duration, the specific trend of each country (controlled in the present study) was able to absorb the influence of this variable on the mortality rate of LICs, since this trend shows a greater slope than in the case of HICs (see Fig. 3). At any rate, the implementation of a DPS system is seen to reduce mortality in HICs.

#### Mean annual precipitation (Precipit)

An inverse effect for mean annual precipitation on the fatality rate is found only in LICs, and with a low statistical significance. Although this effect of negative sign has been previously reported (Calvo-Poyo et al. 2020), the mechanisms by which it can influence different countries with different signs have not been identified (Bergel-Hayat et al. 2013).

#### Fixed effects and country-specific linear trend

**Fig. 2:**
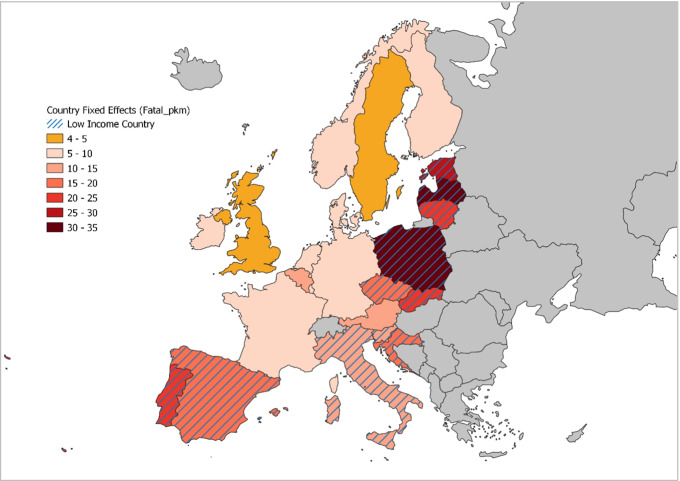
Country-specific fixed effects

The map in Fig. 2 indicates the specific and differential characteristics of each country that are not reflected in the models, through the coefficients of the fixed effects corresponding to each country. This figure clearly shows how the countries in the HIC group have certain characteristics that link them to fixed-effects coefficients that are lower than those of the LICs, hence lower mortality rates. Better national conditions may be attributed to various factors not explicitly considered in this study, e.g., better health care, a greater road safety culture of the population, or higher quality of road infrastructure and vehicles. At the same time, the most peripheral countries within the LICs (Eastern countries and Portugal) are found to show the largest fixed effects, and therefore the most negative characteristics not considered in the generic model with respect to the study problem.

**Fig. 3:**
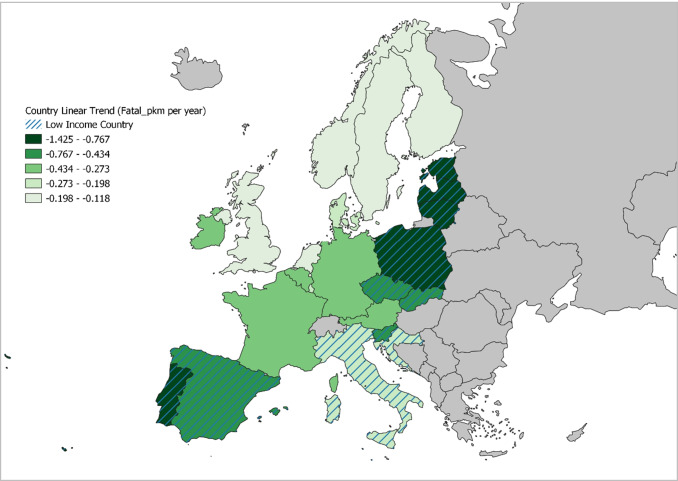
Country-specific linear trend

The differences observed in Fig. 2 suggest greater room for improvement among LICs. Consequently, the LICs generally exhibit a much stronger tendency to reduce their mortality figures than the HICs, as reflected in Fig. 3. Once again, the greatest downward trend in the mortality rate is observed in the peripheral countries of the LICs.

## Conclusions

This article has analyzed how the effect of economic resources invested in roads on mortality can vary according to a given country’s economic development. In addition, it quantifies the impact caused by specific characteristics of each country and the trend in the fatality rate of each country. To this end, the 23 European countries were classified into two groups according to their per capita income: high-income countries (Austria, Belgium, Denmark, Finland, France, Germany, Ireland, Luxembourg, Netherlands, Norway, Sweden, and United Kingdom) and low-income countries (Croatia, Czechia, Estonia, Italy, Latvia, Lithuania, Poland, Portugal, Slovakia, Slovenia, and Spain). For comparative purposes, for each group of countries, panel data models with fixed effects were performed for the period from 1998 to 2016. Due to the complexity of the subject of study, which is influenced by a multitude of aspects, further variables were incorporated to help control for certain factors related to the road network, or socioeconomic, legislative, and meteorological characteristics.

As for the main objective of the study, the two target variables used to control the effects of economic resources on road mortality—investment in construction and expenditure on maintenance—gave significant results. Specifically, it was found that both investment in road construction (in the case of LICs) and maintenance expenditure (in both groups) have a reducing effect on the fatality rate.

It was also found that the extension of the motorway network contributes to the reduction of fatalities in the LICs, that unemployment is associated with reductions in the mortality rate (this effect being much greater in the LICs than in the HICs), and that there is more sensitivity to economic cycles in the LICs: a greater influence of the unemployment rate on the reduction of fatalities and a direct relationship between the annual increase in GDP per capita and mortality. For the HICs group, the annual variation in alcohol consumption influenced the increase in the number of fatalities, whereas the implementation of a point-based license system showed a reducing effect.

Regarding the national conditions reflected by the fixed-effects coefficients of each country, it was found that HICs have much more favorable frameworks for achieving low road mortality rates. In turn, the factors that contribute to increasing this rate appear to be more present in the peripheral countries. Finally, the trend by country indicates a greater potential for improvement in the fatality rate among LICs, particularly in peripheral countries. To sum up, a greater potential for reducing the mortality rate in the long-term is envisaged when the starting fatality rate of the country in question is higher, a situation that tends to occur in the so-called peripheral countries of the European Union.

This study points to evidence that can help policy-makers in their effort to reduce road death figures when attending and allocating the economic resources needed for road infrastructures. More specifically, the group of countries with the lowest economic development within the European context is seen to have the greatest potential for using economic resources as a tool for improving road safety. In this group of countries, investments in road construction and maintenance expenditure are associated with a decrease in road mortality figures. Accordingly, budgeting for them stands as a practical measure to meet specific road safety objectives. Extension of the motorway network also shows a mortality-reducing effect in the short-term. Yet for the group of countries with the highest economic development, the resources allocated to road maintenance are found to yield a beneficial effect on the reduction of road mortality. Another practical application arises from the identification of country-specific characteristics. Namely, lower-income European countries possess a greater potential for road safety improvements.

The results put forth here provide new evidence of the need to consider the socioeconomic level of a country when assessing the impact that road investment and maintenance expenditure might have on road safety. It has been shown how eight independent variables influence this problem depending on the country’s wealth. In addition to country-specific characteristics, a considerable difference exists between central and peripheral countries. Peripheral countries have the most room for improvement regarding road safety problems. Furthermore, the beneficial influence of road construction investment on road safety appears to change at a certain point of economic development. Future work should attempt to establish when this turning point occurs, and investigate the possible existence of a U-curve relationship between road investment and traffic fatalities. Undertaking a similar study in another geographical area, with different socioeconomic conditions, may yield valuable new insights.

## Data Availability

The data that support the findings of this study are available from the corresponding author, J.N.-M., upon reasonable request.

## Appendix

**Table 8:** Extended results for high-income countries (HIC)

	High-income countries					
	(1)	(2)	(3)	(4)	(5)	(6)
	GLS-Parks	GLS-Parks	GLS-Parks	GLS-Parks	GLS-Parks	GLS Driscoll-Kraay SE
Road_inv	.006	.023***	.005	.005	.01**	.019*
	(.006)	(.006)	(.006)	(.006)	(.005)	(.011)
L1. Road_inv	.008	.005	.014**	.027***	.002	−.005
	(.006)	(.006)	(.006)	(.006)	(.005)	(.011)
Road_maint	−.056***	−.081***	−.078***	−.107***	−.012	.016
	(.013)	(.01)	(.012)	(.014)	(.012)	(.027)
L1. Road_maint	−.075***	−.112***	−.098***	−.11***	−.02*	−.053**
	(.014)	(.01)	(.012)	(.014)	(.01)	(.024)
D.Prop_motorwa		−.052	.305	.092	−.316***	−.447
		(.191)	(.206)	(.203)	(.102)	(.608)
D.Mot_index		.011***	.008***	.01***	.003***	.003
		(.001)	(.002)	(.002)	(.001)	(.002)
Unemploy			−.198***	−.25***	−.121***	−.115***
			(.024)	(.023)	(.015)	(.023)
D.GDP_Cap			.005	.008	−.006	−.001
			(.009)	(.006)	(.004)	(.011)
D.Alcohol				.159***	.139***	.17***
				(.021)	(.015)	(.048)
DPS				−1.048***	−.804***	−1.012***
				(.097)	(.075)	(.189)
Precipit				0	0	0
				(0)	(0)	(0)
*United Kingdom (Reference)*						
*Austria*					7.923***	7.863***
					(.26)	(.549)
*Belgium*					6.386***	6.51***
					(.313)	(.624)
*Denmark*					3.302***	3.332***
					(.308)	(.275)
*Finland*					2.38***	2.391***
					(.265)	(.578)
*France*					4.331***	4.254***
					(.517)	(.99)
*Germany*					3.165***	3.189***
					(.226)	(.522)
*Ireland*					4.527***	4.553***
					(.234)	(.523)
*Luxembourg*					4.27***	4.192***
					(.438)	(.952)
*Netherlands*					1.097***	1.29***
					(.324)	(.323)
*Norway*					1.617***	1.53***
					(.17)	(.343)
*Sweden*					.485***	.554
					(.148)	(.406)
*Year (Lineal)*					−.113***	−.118***
					(.011)	(.023)
*Year##United Kingdom (Reference)*						
*Year##Austria*					−.322***	−.306***
					(.024)	(.043)
*Year##Belgium*					−.222***	−.235***
					(.026)	(.031)
*Year##Denmark*					−.112***	−.092***
					(.031)	(.022)
*Year##Finland*					−.082***	−.079*
					(.024)	(.04)
*Year##France*					−.171***	−.183**
					(.047)	(.065)
*Year##Germany*					−.177***	−.184***
					(.021)	(.039)
*Year##Ireland*					−.166***	−.159***
					(.02)	(.017)
*Year##Luxembourg*					−.161***	−.141**
					(.039)	(.055)
*Year##Netherlands*					−.006	−.004
					(.031)	(.027)
*Year##Norway*					−.065***	−.045
					(.02)	(.039)
*Year##Sweden*					−.036***	−.04**
					(.011)	(.018)
_cons	5.127***	5.509***	6.475***	7.549***	4.531***	4.329***
	(.331)	(.183)	(.351)	(.301)	(.232)	(.736)
Observations	216	216	216	216	216	216
*R* ^2^ (within)	-	-	-	-	-	0.9625

Standard errors are in parentheses. “L1” means 1-year lag and “D” means that the series are differentiated

****p*<.01, ***p*<.05, **p*<.1

Autocorrelation parameters for model (1)

**Table:**

*United Kingdom*: 0.8859139847	*Austria*: 0.8643408802054725	*Belgium*: 0.8931951680567731
*Denmark*: 0.8091324928662396	*Finland*: 0.8353460978984687	*France*: 0.8322425414664197
*Germany*: 0.7917206263380858	*Ireland*: 0.8520198140129159	*Luxembourg*: 0.7194550406814
*Netherlands*: 0.9085817615116	*Norway*: 0.6668197205366204	*Sweden*: 0.9276435635378208

Autocorrelation parameters for model (2)

**Table:**

*United Kingdom*: 0.8702445008	*Austria*: 0.8753295399892833	*Belgium*: 0.8998299924556952
*Denmark*: 0.8133310321559241	*Finland*: 0.8449777896347677	*France*: 0.8199047834558654
*Germany*: 0.5257562713198277	*Ireland*: 0.8522079806424541	*Luxembourg*: 0.6650852887522
*Netherlands*: 0.9127556438865	*Norway*: 0.6750694298014217	*Sweden*: 0.913019658454313

Autocorrelation parameters for model (3)

**Table:**

*United Kingdom*: 0.8120501164	*Austria*: 0.8508329312592112	*Belgium*: 0.8807073441937096
*Denmark*: 0.7723068439746752	*Finland*: 0.7846559025005767	*France*: 0.7327709637831794
*Germany*: 0.8275224665620146	*Ireland*: 0.6285615224397949	*Luxembourg*: 0.6326505654864
*Netherlands*: 0.9187699145561	*Norway*: 0.6530329675544595	*Sweden*: 0.8967980998305315

Autocorrelation parameters for model (4)

**Table:**

*United Kingdom*: 0.8226914754	*Austria*: 0.8492717664498125	*Belgium*: 0.8655764270134819
*Denmark*: 0.8486365574724212	*Finland*: 0.7860273723648568	*France*: 0.7374697085879945
*Germany*: 0.8406801443360326	*Ireland*: 0.5422546115641053	*Luxembourg*: 0.5929432994276
*Netherlands*: 0.8348261960175	*Norway*: 0.468879805874015	*Sweden*: 0.910918227463908

Autocorrelation parameters for model (5)

**Table:**

*United Kingdom*: 0.3717345011	*Austria*: 0.1811352030600173	*Belgium*: 0.3940218828472441
*Denmark*: 0.2791679573945999	*Finland*: 0.1831490248202158	*France*: 0.7527308724466616
*Germany*: 0.5058983292237634	*Ireland*: 0.0809960446713882	*Luxembourg*: 0.2259550689788
*Netherlands*: 0.4272479554905	*Norway*: -0.3827231626134385	*Sweden*: 0.1718540682012163

**Table 9:** Extended results for low-income countries (LIC)

	Low-income countries					
	(1)	(2)	(3)	(4)	(5)	(6)
	GLS-Parks	GLS-Parks	GLS-Parks	GLS-Parks	GLS-Parks	GLS Driscoll-Kraay SE
Road_inv	−.003	−.002	−.003	−.009**	−.011***	−.017***
	(.003)	(.003)	(.002)	(.004)	(.002)	(.004)
L1. Road_inv	.006**	.005*	.007***	.009**	.003	−.007
	(.002)	(.003)	(.002)	(.004)	(.002)	(.005)
Road_maint	−.033***	−.039***	−.05***	−.02**	0	−.002
	(.009)	(.009)	(.009)	(.008)	(.006)	(.013)
L1. Road_maint	−.029***	−.032***	−.045***	−.048***	−.02***	−.022**
	(.01)	(.009)	(.01)	(.008)	(.006)	(.01)
D.Prop_motorwa		.034	−.122	−.567**	−.614***	−.83**
		(.199)	(.175)	(.256)	(.184)	(.349)
D.mot_index_1000		.009**	.005**	.001	−.003	.002
		(.003)	(.003)	(.003)	(.003)	(.005)
Unemploy			−.035	−.025	−.133***	−.188**
			(.033)	(.035)	(.028)	(.066)
D.GDP_Cap			.201**	.196**	.301***	.473**
			(.087)	(.095)	(.07)	(.173)
D.Alcohol				.189***	−.053	−.14
				(.07)	(.045)	(.082)
DPS				−.809***	−.228	−.653
				(.246)	(.187)	(1.163)
Precipit				−.003***	−.001***	−.001*
				(0)	(0)	(.001)
*Italy (reference)*						
*Croatia*					4.974***	4.812***
					(.871)	(1.361)
*Czechia*					6.387***	5.074***
					(.653)	(1.442)
*Estonia*					16.092***	14.617***
					(1.089)	(2.166)
*Latvia*					24.321***	23.149***
					(1.766)	(2.438)
*Lithuania*					9.944***	8.987***
					(1.404)	(2.274)
*Poland*					19.342***	18.763***
					(.911)	(1.934)
*Portugal*					9.646***	12.166***
					(.918)	(.874)
*Slovakia*					9.901***	9.6***
					(.825)	(1.608)
*Slovenia*					3.579***	2.908**
					(.596)	(1.022)
*Spain*					8.699***	7.776***
					(.717)	(1.378)
*Year (Linear)*					−.213***	−.2*
					(.039)	(.11)
*Year##Italy (reference)*						
*Year##Croatia*					−.156**	−.181
					(.073)	(.118)
*Year##Czechia*					−.3***	−.276***
					(.041)	(.063)
*Year##Estonia*					−1.136***	−1.13***
					(.096)	(.172)
*Year##Latvia*					−1.244***	−1.225***
					(.167)	(.146)
*Year##Lithuania*					−.552***	−.574***
					(.129)	(.138)
*Year##Poland*					−.992***	−1.052***
					(.072)	(.145)
*Year##Portugal*					−.666***	−.842***
					(.072)	(.09)
*Year##Slovakia*					−.505***	−.557***
					(.06)	(.119)
*Year##Slovenia*					−.298***	−.298**
					(.044)	(.107)
*Year##Spain*					−.478***	−.413***
					(.055)	(.067)
_cons	9.794***	9.932***	10.42***	11.921***	9.448***	11.589***
	(.719)	(.74)	(.554)	(.707)	(.711)	(1.456)
Observations	198	198	198	198	198	198
*R*^2^ (within)	-	-	-	-	-	0.9464

Standard errors are in parentheses. “L1” means 1-year lag and “D” means that the series are differentiated

****p*<.01, ***p*<.05, **p*<.1

Autocorrelation parameters for model (1)

**Table:**

*Italy*: 0.8355485854590473	*Croatia*: 0.8073574324335875	*Czechia*: 0.8180890295726044
*Estonia*: 0.7346387958096933	*Latvia*: 0.8817888364606743	*Lithuania*: 0.821738996779305
*Poland*: 0.8948837625479206	*Portugal*: 0.6806908347788688	*Slovakia*: 0.7487801887374294
*Slovenia*: 0.9331336424357719	*Spain*: 0.8741474205089699	

Autocorrelation parameters for model (2)

**Table:**

*Italy*: 0.8092638215698561	*Croatia*: 0.8034184817590622	*Czechia*: 0.8132071420698803
*Estonia*: 0.7459641846012741	*Latvia*: 0.8760764953055938	*Lithuania*: 0.524188707680019
*Poland*: 0.8905775770055319	*Portugal*: 0.6790643360497832	*Slovakia*: 0.7619297045293975
*Slovenia*: 0.9323756457675452	*Spain*: 0.8625680761709628	

Autocorrelation parameters for model (3)

**Table:**

*Italy*: 0.7091470593115121	*Croatia*: 0.7708131308549094	*Czechia*: 0.5980719635075309
*Estonia*: 0.7103861014868622	*Latvia*: 0.8602638737115452	*Lithuania*: 0.536610875985165
*Poland*: 0.8372787684338128	*Portugal*: 0.7160844165779018	*Slovakia*: 0.7152680910085032
*Slovenia*: 0.8951045277391969	*Spain*: 0.8521178306501836	

Autocorrelation parameters for model (4)

**Table:**

*Italy*: 0.548800379800658	*Croatia*: 0.5082779633614529	*Czechia*:0.6630145724673687
*Estonia*: 0.7675305339584214	*Latvia*: 0.8609453291750576	*Lithuania*: 0.662307908062095
*Poland*: 0.8604010118271381	*Portugal*: 0.7709948986517203	*Slovakia*: 0.7510747807946062
*Slovenia*: 0.35882392525338	*Spain*: 0.8035554765147533	

Autocorrelation parameters for model (5)

**Table:**

*Italy*: 0.1445825795993902	*Croatia*: 0.2874130918812479	*Czechia*: -0.2368250253681359
*Estonia*: 0.0444888594695389	*Latvia*: 0.4302654871426296	*Lithuania*: 0.573041187452992
*Poland*: 0. 1438385592075945	*Portugal*: 0.5051358032991847	*Slovakia*: -0.048284112232479
*Slovenia*: 0.3868086267405818	*Spain*: 0.5073272977453901	

**Table 10:** Country abbreviations

Austria	*AT*	Belgium	*BE*	Croatia	*HR*
Czechia	*CZ*	Denmark	*DK*	Estonia	*EE*
Finland	*FI*	France	*FR*	Germany	*DE*
Ireland	*IE*	Italy	*IT*	Latvia	*LV*
Lithuania	*LT*	Luxembourg	*LU*	Netherlands	*NL*
Norway	*NO*	Poland	*PL*	Portugal	*PT*
Slovakia	*SK*	Slovenia	*SI*	Spain	*ES*
Sweden	*SE*	United Kingdom	*UK*		

**Table 11:** Alcohol consumption in high-income countries (in liters per capita)

	*AT*	*BE*	*DK*	*FI*	*FR*	*DE*	*IE*	*LU*	*NL*	*NO*	*SE*	*UK*
*1998*	12.90	9.92	11.69	8.60	13.27	12.74	14.44	12.88	9.93	5.24	6.80	10.14
*1999*	12.80	10.10	11.62	8.62	13.15	12.94	14.95	12.92	10.06	5.45	6.88	10.16
*2000*	13.20	11.25	11.68	8.59	13.63	12.91	13.87	13.14	10.06	5.67	6.20	10.82
*2001*	12.40	11.05	11.55	8.94	13.89	12.46	14.06	12.89	9.95	5.49	6.60	11.29
*2002*	12.50	11.33	11.33	9.25	13.78	12.25	13.98	12.91	9.68	5.89	6.90	11.31
*2003*	12.20	11.31	11.54	9.31	13.49	11.92	13.07	12.61	9.56	6.04	6.90	11.25
*2004*	12.10	12.06	11.27	9.89	13.18	11.83	13.14	12.42	9.56	6.22	6.60	11.55
*2005*	12.40	12.21	11.27	9.95	12.60	12.04	13.42	12.02	9.67	6.37	6.50	11.37
*2006*	12.40	10.94	11.02	10.15	12.40	12.14	13.38	12.17	9.78	6.47	6.50	10.96
*2007*	12.50	13.43	11.00	10.45	12.60	11.89	13.37	11.94	9.51	6.60	6.90	11.14
*2008*	12.00	10.51	10.71	10.26	12.30	11.75	12.42	11.72	9.61	6.75	6.90	10.78
*2009*	11.30	10.10	10.09	9.96	12.30	11.61	11.27	11.60	9.22	6.68	7.30	10.14
*2010*	12.10	10.27	10.24	9.72	12.33	11.35	11.63	11.72	9.32	6.59	7.31	10.22
*2011*	11.90	10.14	10.22	9.81	12.37	11.87	11.65	12.01	9.17	6.44	7.34	10.02
*2012*	12.10	10.09	9.10	9.27	12.24	11.76	11.53	11.89	9.28	6.21	7.23	9.76
*2013*	11.60	10.33	9.43	9.07	11.64	11.67	10.64	11.55	8.54	6.22	7.32	9.65
*2014*	12.20	10.57	9.53	8.75	11.97	11.60	11.00	11.69	8.16	6.06	7.20	9.69
*2015*	11.40	10.36	9.38	8.51	11.87	11.99	10.93	11.83	8.03	5.97	7.16	9.82
*2016*	11.70	9.42	9.55	8.43	11.74	10.90	11.46	11.22	8.29	6.03	7.18	9.81

**Table 12:** Alcohol consumption in low-income countries (in liters per capita)

	*HR*	*CZ*	*EE*	*IT*	*LV*	*LT*	*PL*	*PT*	*SK*	*SI*	*ES*
*1998*	13.84	13.92	8.01	8.98	8.94	6.35	8.32	12.48	12.13	14.61	11.58
*1999*	12.15	13.85	8.85	8.86	8.84	6.75	8.22	13.18	12.13	12.28	11.33
*2000*	14.06	13.98	7.90	9.78	7.13	9.87	8.40	13.08	11.06	12.80	11.84
*2001*	14.56	13.75	9.22	9.68	6.68	10.20	7.74	13.41	10.73	11.58	12.35
*2002*	14.83	13.72	10.36	9.25	7.44	11.00	8.02	13.23	10.78	9.87	10.74
*2003*	13.80	13.88	11.55	9.30	8.24	11.29	9.06	14.21	9.85	11.55	11.35
*2004*	13.12	13.31	13.23	8.98	8.81	12.10	9.19	13.47	10.03	10.01	11.68
*2005*	11.58	13.26	14.70	7.41	9.92	12.30	9.50	13.34	10.83	11.19	11.92
*2006*	11.84	13.12	15.83	7.26	10.40	12.70	10.40	13.12	10.31	12.26	11.86
*2007*	12.28	13.35	17.37	7.19	12.12	13.40	10.90	12.59	10.63	11.02	11.05
*2008*	12.30	13.35	16.38	6.84	11.84	13.30	11.40	12.35	11.43	10.94	10.24
*2009*	12.07	13.31	14.32	6.40	9.85	12.40	10.70	12.03	10.69	10.52	9.99
*2010*	12.11	12.65	14.97	6.95	9.83	13.61	10.04	12.23	10.55	10.31	9.78
*2011*	12.43	12.61	16.27	6.98	10.11	14.88	10.21	11.91	10.78	10.61	9.01
*2012*	11.92	12.97	16.96	7.48	10.20	15.15	10.19	10.86	10.66	10.95	8.83
*2013*	11.64	12.84	17.75	7.35	10.43	15.14	10.79	10.54	10.54	9.53	8.77
*2014*	10.64	13.06	17.29	7.56	10.60	14.82	10.45	10.35	10.89	10.92	8.70
*2015*	9.89	12.82	16.64	7.14	10.82	14.42	10.48	10.54	10.78	11.49	8.26
*2016*	10.32	12.99	15.35	7.08	11.19	13.61	10.43	10.66	10.14	10.51	8.58
